# Omega-3 Fatty Acids Attenuate Renal Fibrosis via AMPK-Mediated Autophagy Flux Activation

**DOI:** 10.3390/biomedicines11092553

**Published:** 2023-09-17

**Authors:** Suyeon Han, Hyunsu Choi, Hyerim Park, Jwa-Jin Kim, Eu-Jin Lee, Young-Rok Ham, Ki-Rayng Na, Kang-Wook Lee, Yoon-Kyung Chang, Dae-Eun Choi

**Affiliations:** 1 Department of Nephrology, Chungnam National University Hospital, Daejeon 35015, Republic of Korea; garlic1208@cnuh.co.kr (S.H.); eujinlee@cnuh.co.kr (E.-J.L.); youngrok01@cnuh.co.kr (Y.-R.H.); drngr@cnu.ac.kr (K.-R.N.); kwlee@cnu.ac.kr (K.-W.L.); 2 Clinical Research Institute, Daejeon Saint Mary’s Hospital, Daejeon 34943, Republic of Korea; peace420@cmcdj.or.kr; 3 Department of Medical Science, Medical School, Chungnam National University, Daejeon 35015, Republic of Korea; hye05240@gmail.com (H.P.); kjj4827@gmail.com (J.-J.K.); 4 Department of Nephrology, Daejeon Saint Mary’s Hospital, Catholic University of Korea, Daejeon 34943, Republic of Korea

**Keywords:** ω3-PUFA, autophagy flux, UUO, renal fibrosis

## Abstract

The unilateral ureteral obstruction (UUO) injury model is well-known to mimic human chronic kidney disease, promoting the rapid onset and development of kidney injury. ω3-poly unsaturated fatty acids (PUFAs) have been observed to protect against tissue injury in many disease models. In this study, we assessed the efficacy of ω3-PUFAs in attenuating UUO injury and investigated their mechanism of action. The immortalized human proximal tubular cells human kidney-2 (HK2) were incubated for 72 h with docosahexaenoic acid (DHA) or eicosapentaenoic acid (EPA) in various concentrations, in the presence or absence of transforming growth factor (TGF)-β. DHA/EPA reduced the epithelial–mesenchymal transition in the TGF-β-treated HK2 cells by enhancing autophagy flux and adenosine monophosphate-activated protein kinase (AMPK) phosphorylation. C57BL/6 mice were divided into four groups and treated as follows: sham (no treatment, *n* = 5), sham + ω3-PUFAs (*n* = 5), UUO (*n* = 10), and UUO + ω3-PUFAs (*n* = 10). Their kidneys and blood were harvested on the seventh day following UUO injury. The kidneys of the ω3-PUFAs-treated UUO mice showed less oxidative stress, inflammation, and fibrosis compared to those of the untreated UUO mice. Greater autophagic flux, higher amounts of microtubule-associated protein 1A/1B-light chain 3 (LC3)-II, Beclin-1, and Atg7, lower amounts of p62, and higher levels of cathepsin D and ATP6E were observed in the kidneys of the omega-3-treated UUO mice compared to those of the control UUO mice. In conclusion, ω3-PUFAs enhanced autophagic activation, leading to a renoprotective response against chronic kidney injury.

## 1. Introduction

Chronic kidney disease (CKD) is a progressive disease that leads to renal failure and may cause patients to require renal replacement therapy. The number of patients with CKD has increased in the past few decades, resulting in an increased burden of CKD globally [1]. The loss of renal function in CKD patients is mainly due to the development of fibrosis, including glomerular sclerosis and tubulointerstitial fibrosis [2]. The unilateral ureteral obstruction (UUO) model is an experimental rodent model that enables the study of obstructive renal injury. It closely mimics human chronic kidney disease, promoting the rapid onset and development of kidney injury [3]. In the UUO model, mechanical stretching leads to epithelial tubular cell apoptosis, oxidative stress, and inflammation, which cause progressive renal failure and tubulointerstitial fibrosis [4,5].

The role of autophagy in renal fibrosis—the main pathophysiological mechanism of CKD—is disputed. Livingstone et al. reported that the inhibition of autophagy resulted in the suppression of renal tubulointerstitial fibrosis in UUO ATG7 knockout mice [6]. The persistent autophagy activation observed in mouse kidneys subjected to a nephrotoxic treatment consisting of repeated low doses of cisplatin is known to promote renal fibrosis and chronic kidney disease [7]. However, an emerging body of evidence indicates that autophagy plays a protective role against CKD progression. A protective role of autophagy against renal fibrosis was reported in the UUO model [8,9]. In these mice, autophagy was shown to regulate the expression of transforming growth factor (TGF)-β and to inhibit renal tubulointerstitial fibrosis [10]. When mice carrying a Beclin-1-activating mutation were subjected to UUO, autophagy was enhanced, which led to a reduction in renal fibrosis [11].

ω3-polyunsaturated fatty acids (ω3-PUFAs) are essential fatty acids for humans and are present in various foods [12]; in particular, they are abundant in marine food, such as fish roe, shrimp, and shellfish [13]. It is known that ω3-PUFAs exert protective effects against cardiovascular injuries, including brain, liver, and heart injuries [14,15,16]. Several studies have attributed the protective effect of ω3-PUFAs to their antioxidant properties [17,18,19].

We reported that ω3-PUFAs derived from ω6-PUFAs attenuated acute renal injury caused by renal ischemia/reperfusion by enhancing autophagy activation via AMP-activated protein kinase (AMPK) [20]. In this study, we aimed to verify that ω3-PUFAs exerted their reno-protective effect via autophagy flux enhancement. To this end, we evaluated autophagy flux by examining the levels of autophagic substrates involved in each autophagic step using immunofluorescence microscopy.

## 2. Materials and Methods

### 2.1. Cell Culture and Drug Treatment

Human kidney-2 (HK-2) cells, an immortalized human PTC line, purchased from American Type Culture Collection (Mannasas, VA, USA), were grown in Dulbecco’s Modified Eagle Medium (DMEM)/F12 (Gibco, New York, NY, USA). The medium was enriched with 15 mM N-2-hydroxyethylpiperazine-N-2-ethanesulfonic acid (HEPES) (Sigma-Aldrich, St. Louis, MO, USA), pyridoxine HCl (Gibco, NY, USA), L-glutamine (Sigma-Aldrich, MO, USA), and 10% (*v*/*v*) fetal calf serum (Gibco, NY, USA). The cells were incubated at 37 °C in 95% humidified air and 5% CO_2_ with various concentrations of the ω3-PUFAs such as eicosapentaenoic acid (EPA) and docosahexaenoic acid (DHA) (Sigma-Aldrich, St. Louis, MO, USA) in the presence or absence of TGF-β1 for 72 h.

### 2.2. Animal Model

C57BL/6 mice (10 weeks old, male) were obtained from SAMTAKO Bio Korea (Gyounggido, Republic of Korea). The mice were housed in an environment with a 12-h light/12-h dark cycle. The mice had unrestricted access to food and water. All the animal experiments were carried out with the approval of the Animal Use and Care Committee at the Catholic University of Korea Daejeon St. Mary’s Hospital (CMCDJ-AP-2014-001, 26 January 2014). The mice were divided into 4 groups: wild-type (wt) sham (*n* = 5), sham + ω3-PUFAs (*n* = 5), UUO (*n* = 10), and UUO + ω3-PUFAs (*n* = 10). UUO injury was performed as described previously [21]. The mice were anesthetized through administration of an intraperitoneal injection of ketamine (60 mg/kg body mass) and xylazine (8 mg/kg). The left ureter mid portion was ligated using 6-0 silk, following an abdominal incision. Then, 2 g/kg of ω3-PUFAs (United Pharmaceutical, Seoul, Republic of Korea) was administered daily orally using zonde from the day before UUO to the 6th day following UUO. The mice were sacrificed on the seventh day following UUO injury, and their blood and kidneys were harvested.

### 2.3. Tissue Preparation

The tissues were prepared in accordance with the method previously described by Jeong J.Y. et al [21]. The left UUO kidney was collected immediately after euthanasia and was split into three portions. Two segments were rapidly frozen in liquid nitrogen and kept at −70 °C for later RNA and protein extraction. The remaining section of the kidney was preserved in 10% buffered formaldehyde at room temperature and subsequently embedded in Paraplast (Sherwood Medical, St. Louis, MO, USA) for light microscopy examination.

### 2.4. Tubulo-Interstitial Injury Score

The kidney tissue was embedded in paraffin blocks, sliced into 4 µm sections, and affixed to glass slides. These sections underwent deparaffinization using xylene, followed by staining with hemotoxylin and eosin (H&E), and were observed using an Olympus BX51 microscope (Olympus, Tokyo, Japan). Five consecutive fields were examined at 200× magnification, and an average score for tissue injury was calculated per slide. The assessment and scoring of renal cortical vacuolization, proximal tubule simplification, and peritubular/proximal tubule leukocyte infiltration in the H&E sections was performed, assigning the scores as follows: 0, no injury; 1, <25% injury; 2, 26–50% injury; 3, 51–75% injury, 4, and >75% injury. An experienced pathologist evaluated the injuries in the H&E-stained section and attributed the scores in a blind fashion.

### 2.5. Western Blot Analysis

Kidney tissues and cell samples were homogenized in RIPA (radioimmunoprecipitation assay) buffer (Elpis Biotech., Daejeon, Republic of Korea) containing a protease inhibitor cocktail (Roche Diagnostics GmbH, Mannheim, Germany). The protein concentrations were determined using a BCA (bicinchoninic acid) protein assay kit (Thermo Scientific, Rockford, IL) in accordance with the manufacturer’s instructions. Twenty micrograms of total protein from each tissue or cell lysate sample was separated by gel electrophoresis and then transferred to a nitrocellulose membrane (GE Healthcare Bio-Sciences, Piscataway, NJ, USA). After blocking, the membranes were incubated overnight with primary antibodies at 4 °C with gentle shaking. The membranes were then incubated with horseradish peroxidase-conjugated anti-rabbit IgG (Invitrogen, Carlsbad, CA, USA; dilutions 1:5000) or anti-mouse IgG (Invitrogen; dilutions 1:5000). Detection and quantification of the protein bands was performed using the ChemiDoc™ XRS+ (Bio-Rad Laboratories, Hercules, CA, USA). Each membrane was probed with an anti-glyceraldehyde-3-phosphate dehydrogenase (GAPDH) antibody (Cell Signaling Technology, Danvers, MA, USA) as a loading control. Anti-α-smooth muscle actin (SMA) (Abcam, Cambridge, CB2 OAX, UK), anti-E-cadherin (Abcam, Cambridge, CB2 OAX, UK), anti-phospho-AMPKα (Cell Signaling Technology, Danvers, MA, USA), anti-AMPKα (Cell Signaling Technology, Danvers, MA, USA), anti-LC3 (Cell Signaling Technology, Danvers, MA, USA), anti-p62 (Cell Signaling Technology, Danvers, MA, USA), anti-fibronectin (Abcam, Cambridge, CB2 OAX, UK) and anti-collagen IV (Abcam, Cambridge, CB2 OAX, UK) were the primary antibodies used in this study. All primary antibodies were diluted at 1:1000.

### 2.6. Real-Time Polymerase Chain Reaction

Total RNA was extracted using TRIzol reagent (Invitrogen, Carlsbad, CA, USA) in accordance with the manufacturer’s instructions. The Reverse Transcriptase Premix Kit (Elpis Biotech, Daejeon, Republic of Korea) was used to synthesize complementary DNA (cDNA) from 1 mg aliquots of RNA. The cDNA was then used as a template in polymerase chain reactions (PCR) employing gene-specific primer pairs. The amplification of the cDNA was accomplished using the Power SYBR Green PCR Master Mix (Applied Biosystems, Warrington, UK). The ABI 7500 FAST platform (Applied Biosystems, Foster City, CA, USA) was used for quantitative real-time PCR. The relative levels of mRNA were normalized to those of glyceraldehyde-3-phosphate dehydrogenase (GAPDH). The primer sequences were as follows (5′–3′): GAPDH sense: AACTTTGGCATTGTGGAAGG, anti-sense: ACACATTGGGGGTAGGAACA; monocyte chemoattractant protein (MCP)-1 sense: CAATCAATGCCC CAGTCAC, anti-sense: GATTCTTGGGTTGTGGGAGTG; Osteopontin sense: TGAAAGTGACTGATTCTGGCA, anti-sense: GGACGATTGGAGTGAAAGTGT. The results were evaluated using the ΔΔCt method.

### 2.7. Immunohistochemical Staining

Formalin-fixed, paraffin-embedded 4 μm thick sections were deparaffinized and rehydrated. The sections were incubated overnight at 4 °C with 1:200 dilutions of anti-α-SMA, -collagen IV, -fibronectin, -8-Hydroxy-2′-deoxyguanosine (8-OHdG), and -F4/80 (Abcam, Cambridge, CB2 OAX, UK), following antigen retrieval. After multiple phosphate-buffered saline (PBS) washes, a universal biotinylated secondary antibody was applied at concentrations ranging between 1:50 and 1:200. Subsequently, a streptavidin–peroxidase solution was added, following the instructions provided in the Vectastain ABC Kit (Vector Labs, Burlingame, CA, USA). Color development was achieved using a 3,3′-diaminobenzidine (DAB) solution (Vector Labs). The sections were counterstained with hematoxylin, then sequentially rehydrated in various alcohol/water mixtures and xylene before being covered with coverslips. For the negative control, the primary antibody was omitted, and then sections were incubated with the corresponding secondary antibodies and the detection solutions. Immunohistochemical staining evaluation involved calculating the area of stained pixels using the ImageJ software (https://imagej.nih.gov/ij/download.html, accessed on 1 June 2022) in a blinded manner [22].

### 2.8. Immunofluorescent Staining

The levels of Microtubule-associated protein 1 light chain 3 (LC3)-II and lysosomal associated membrane protein (LAMP)-1 were visualized through confocal microscopy following immunofluorescent staining. The cells were exposed to primary antibodies targeting LC3-II (1:400, Medical and Biological Laboratory Co., Ltd., Woods Hole, MA, USA) and LAMP-1 (1:400, Santa Cruz Biotechnology, Dallas, TX, USA). After washing with PBS, the sections were incubated with a 1:500 dilution of Alexa fluor 488-conjugated anti-mouse IgG antibody (Cell signaling) or Alexa fluor 594-conjugated anti-rabbit IgG antibody (1:500 dilution; Cell signaling) for 1 h in the dark at room temperature. Subsequently, the nuclei were stained with 4′,6-diamidino-2-Phenylindole (DAPI) for 5 min, and then the samples were mounted. For the negative control, the primary antibody was omitted, and the sections were incubated with the corresponding secondary antibodies and the detection solutions. A confocal microscope (LSM 700; Zeiss, Jena, Germany) was used to capture fluorescence images Immunofluorescent staining evaluation involved the calculation of mean fluorescence intensity (MFI) using the ImageJ software (https://imagej.nih.gov/ij/download.html, accessed on 1 June 2022) in a blinded manner [6,22,23].

### 2.9. Statistical Analysis

The data are expressed as means ± standard deviations (SDs). One-way ANOVA was used with a post hoc Bonferroni correction to analyze multiple comparisons among the groups. The statistical analysis was carried out using the SPSS software (ver. 20.0 for Windows; SPSS, Inc., Chicago, IL, USA). The differences among the groups were considered significant at *p* < 0.05.

## 3. Results

### 3.1. EPA/DHA Reduced Epithelial–Mesenchymal Transition (EMT) in TGF-β-Treated HK2 Cells

**Figure 1:**
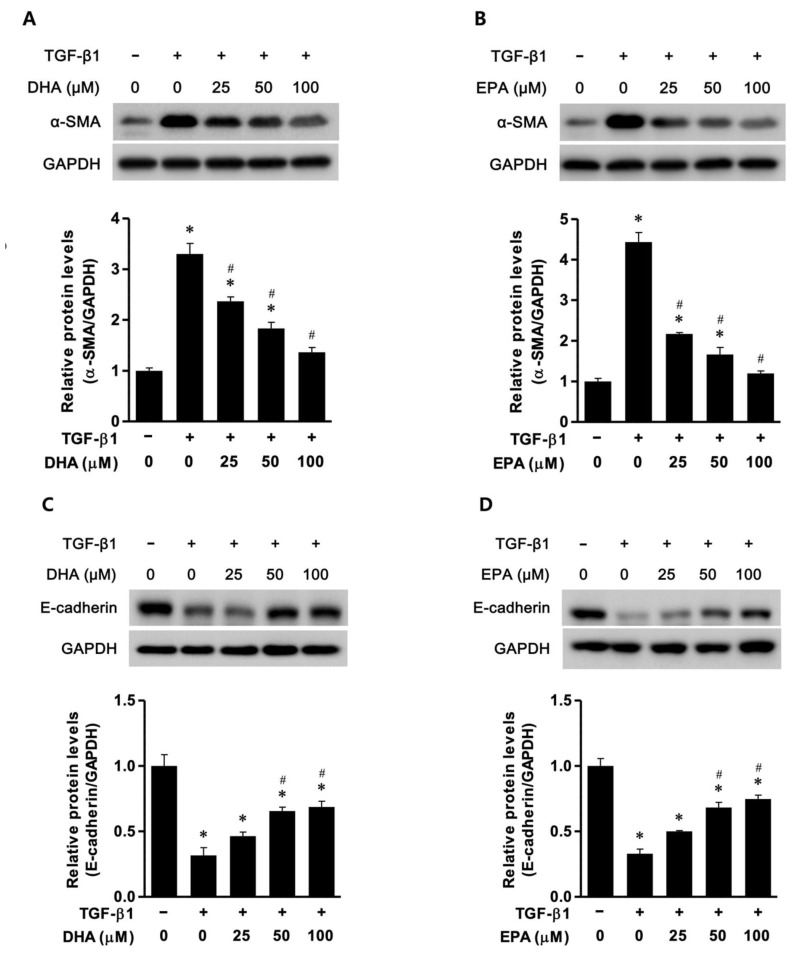
DHA/EPA treatment inhibited the expression of EMT in TGF-β1-treated (10 ng/mL) HK2 cells in a dose-dependent manner. (**A**,**B**) Representative Western blot images: DHA/EPA inhibited the expression of α-SMA in a dose-dependent manner in TGF-β1-treated HK2 cells. (**C**,**D**) Representative Western blot images: DHA/EPA increased E-cadherin levels in a dose-dependent manner in TGF-β1-treated HK2 cells. * *p* < 0.05 vs. control HK2 cells, # *p* < 0.05 vs. TGF-β1-treated HK2 cells in the absence of DHA/EPA. All experiments were performed in triplicate. All values are expressed as the mean ± SD. HK2: human kidney 2; GAPDH: glyceraldehyde-3-phosphate dehydrogenase; EMT: epithelial–mesenchymal transition; DHA: docosahexaenoic acid; EPA: eicosapentaenoic acid; TGF: transforming growth factor; SMA: α-smooth muscle actin.

The levels of E-cadherin and α-SMA were examined to verify the effect of EPA/DHA on the EMT. In the TGF-β-treated (10 ng/mL) HK2 cells, the E-cadherin levels were found to be significantly decreased compared to those in the control HK2 cells. DHA and EPA increased the expression of E-cadherin in the TGF-β-treated HK2 cells in a dose-dependent manner (Figure 1A,B). The levels of α-SMA were found to be significantly increased in the TGF-β-treated HK2 cells compared to the control HK2 cells. However, DHA and EPA induced a reduction in the expression of α-SMA in the TGF-β-treated HK2 cells in a dose-dependent manner (Figure 1C,D). Thus, DHA/EPA upregulated the expression of the epithelial marker E-cadherin in a dose-dependent manner and markedly downregulated the expression of the mesenchymal marker α-SMA in a dose-dependent manner in the TGF-β-treated HK2 cells. DHA and EPA attenuated the renal EMT in the TGF-β-treated HK2 cells.

### 3.2. EPA/DHA Induced Autophagy Flux in TGF-β-Treated HK2 Cells

**Figure 2:**
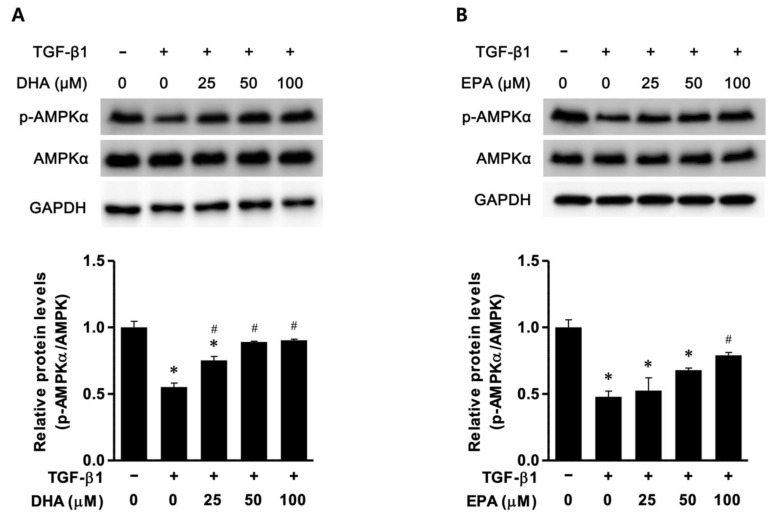

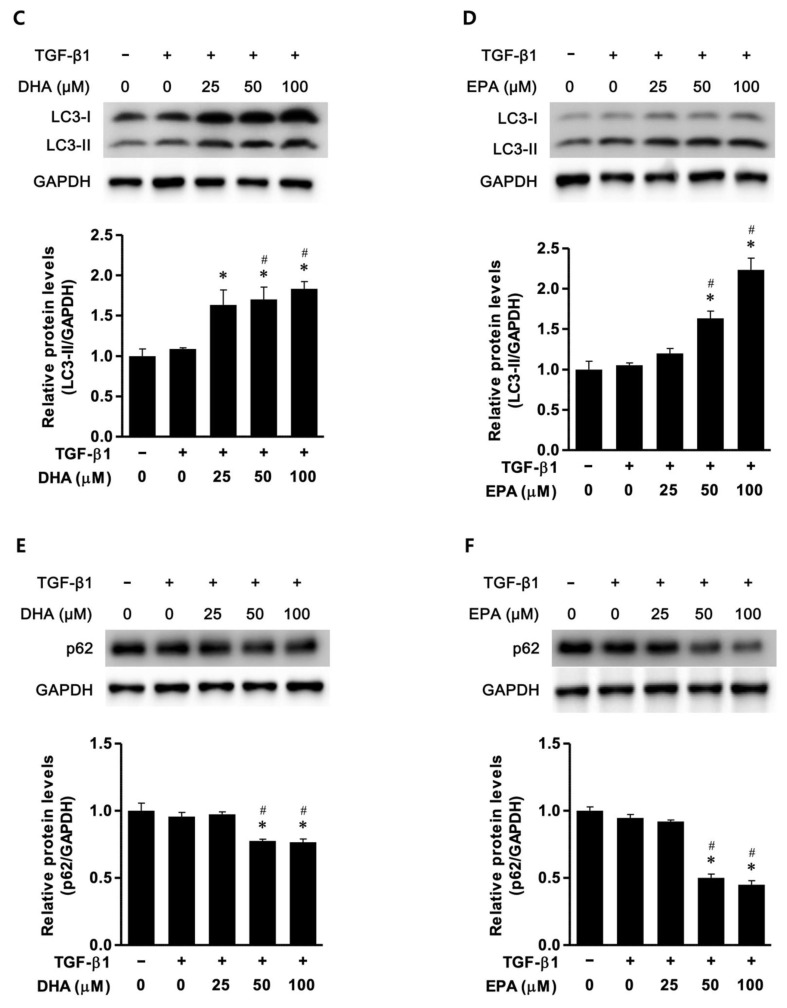
DHA/EPA enhanced autophagic flux via AMPK phosphorylation (**A**,**B**). Representative Western blot images: the p-AMPKα/AMPK ratio was decreased in TGF-β-treated (10 ng/mL) HK2 cells compared to control HK2 cells. DHA/EPA increased the p-AMPKα/AMPKα ratio in TGF-beta-treated HK2 cells, in a dose-dependent manner. (**C**,**D**) Representative Western blot images: DHA/EPA increased the expression of LC3-II in TGF-beta-treated HK2 cells. in dose-dependent manner. (**E**,**F**) Representative Western blot images: DHA/EPA decreased p62 expression in TGF-β1-treated HK2 cells. All experiments were performed in triplicate. All values are expressed as the mean ± SD. * *p* < 0.05 vs. control HK2 cells; # *p* < 0.05 vs. TGF-β1-treated HK2 cells in the absence of DHA/EPA. HK2: human kidney 2; GAPDH: glyceraldehyde-3-phosphate dehydrogenase; EMT: epithelial–mesenchymal transition; DHA: docosahexaenoic acid; EPA: eicosapentaenoic acid; TGF: transforming growth factor; SMA: α-smooth muscle actin.

The phosphorylation of AMPK and the expression of LC3-II and p62 were examined to verify the effect of EPA/DHA on autophagy flux. AMPK phosphorylation was found to be significantly reduced in the TGF-β-treated HK2 cells compared to the control HK2 cells, but was found to increase after the administration of DHA and EPA in a dose-dependent manner (Figure 2A,B). In the TGF-β-treated HK2 cells, the expression of LC3-II was found to be significantly increased compared to that in the control HK2 cells, and further increased in the presence of DHA and EPA in a dose-dependent manner (Figure 2C,D). The expression of p62 was not significantly affected in the TGF-β-treated HK2 cells compared to the control HK2 cells; however, it was reduced in a dose-dependent manner in the presence of DHA and EPA (Figure 2E,F).

### 3.3. Oral Administration of ω3-PUFAs Reduced Renal Oxidative Stress and Inflammation in the Kidneys of Mice Subjected to UUO

**Figure 3:**
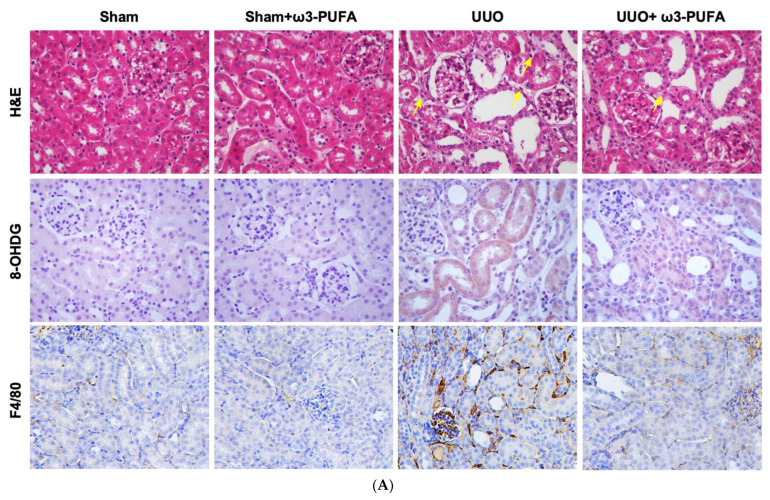

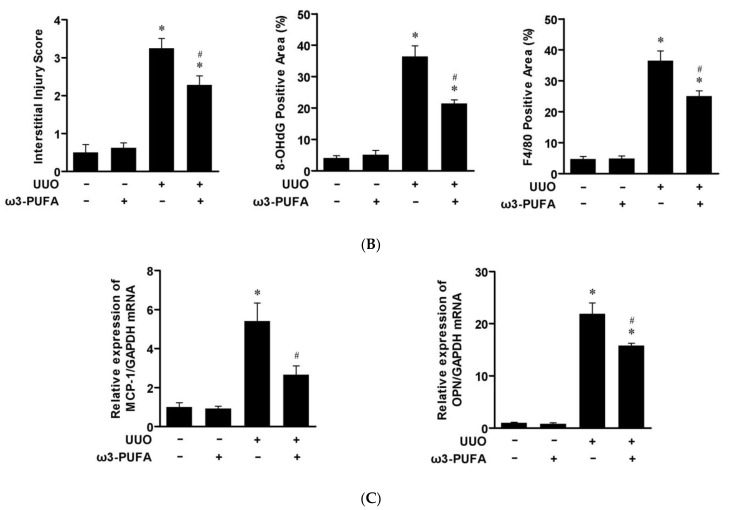
Anti-inflammatory effects of ω3-PUFAs in kidneys subjected to UUO. (**A**) Representative photomicrographs (×200) of renal sections stained with H&E and immunostained for 8-OHDG and F4/80. In the H&E images, infiltration of inflammatory cells (yellow arrows) in the interstitial space is evident. (**B**) Semiquantitative analysis of the interstitial injury score, 8-OHDG-stained area and F4/80-positive cells. (**C**) mRNA expression of MCP-1 and osteopontin. All experiments for mRNA expression evaluation were performed in triplicate. All values are expressed as the mean ± SD. * *p* < 0.05 vs. sham, # *p* < 0.05 vs. UUO-untreated. H&E: hemotoxylin and eosin; 8_OHDG: 8-hydroxy-2′-deoxyguanosine; PUFAs: polyunsaturated fatty acids; UUO: unilateral ureteral obstruction; GAPDH: glyceraldehyde-3-phosphate dehydrogenase; OPN: osteopontin; MCP-1: monocyte chemoattractant protein 1; SD: standard deviation.

Tubular cell damage and interstitial inflammation were found to be significantly increased in the kidneys of the UUO mice compared to those of the sham mice. Based on the interstitial injury scores, the treatment with oral ω3-PUFAs was found to reduce the damage induced by UUO in the kidneys. We performed 8-OHDG staining to evaluate the oxidative stress. We observed that 8-OHDG staining in the damaged kidneys from the UUO-treated mice was significantly increased and that the treatment with oral ω3-PUFAs significantly reduced it. To confirm the effect of ω3-PUFAs on renal inflammation, we focused on F4/80-positive cells. We found that the levels of F4/80-positive cells were significantly increased in the kidneys subjected to UUO compared to the control kidneys, but decreased significantly following oral ω3-PUFAs treatment, thus confirming the anti-inflammatory effect of orally administered ω3-PUFAs on the kidneys (Figure 3A,B). The ω3-PUFAs also decreased the initially elevated mRNA expression of MCP-1 and osteopontin in the kidneys of the UUO-treated mice (Figure 3C). Thus, the oral ω3-PUFAs reduced renal oxidative stress and inflammation in the UUO mice.

### 3.4. The Oral Administration of ω3-PUFAs Reduced Renal Fibrosis in the Kidneys of Mice Subjected to UUO

To verify the effect of the orally administered ω3-PUFAs on renal fibrosis in the UUO mice, Masson’s trichrome staining was performed. In addition, the expression of the fibrosis markers α-SMA, collagen IV, and fibronectin was evaluated through immunohistochemical staining and Western blotting.

**Figure 4:**
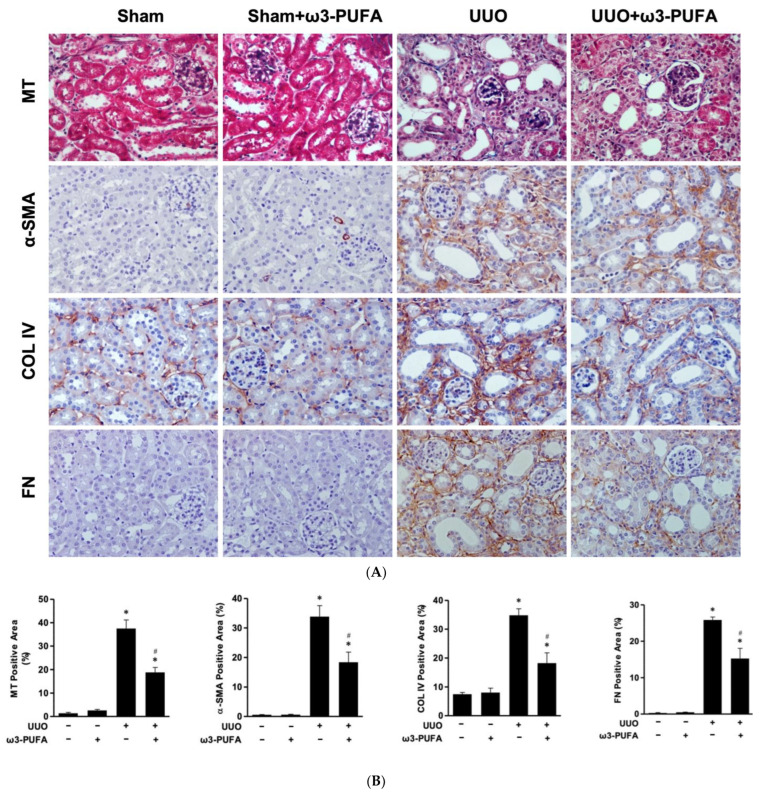

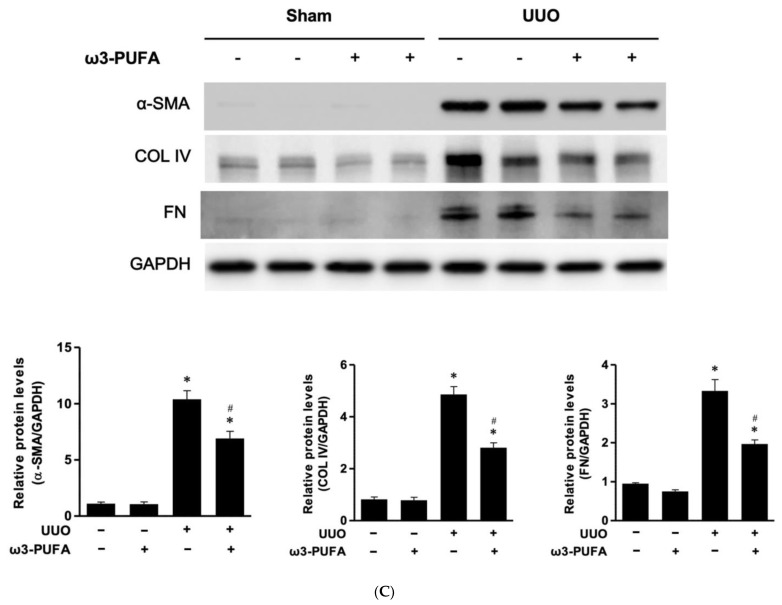
Anti-fibrosis effects of ω3-PUFA on kidneys subjected to UUO. (**A**) Representative photomicrographs (×200) of renal sections stained with Masson’s trichrome stain and immunohistochemical staining of α-SMA, collagen IV, and fibronectin. (**B**) Semiquantitative analysis of the interstitial injury score for α-SMA, collagen IV, and fibronectin-stained areas. (**C**) Representative Western blot images: expression of fibronectin, collagen IV, and a-SMA in the kidneys of sham and UUO mice treated or not with omega-3 PUFA. All Western blots experiments were performed in triplicate. All values are expressed as the mean ± SD. * *p* < 0.05 vs. Sham, # *p* < 0.05 vs. UUO-untreated. MT: Masson’s trichrome stain; COL IV: collagen IV; FN: fibronectin; SMA: smooth muscle actin; PUFAs: polyunsaturated fatty acids; UUO: unilateral ureteral obstruction; GAPDH: glyceraldehyde-3-phosphate dehydrogenase.

Masson’s trichrome staining revealed a significant proportion of blue stained areas in the kidneys of the UUO mice compared to those of the sham mice. The treatment with oral ω3-PUFAs reduced these blue stained areas in the kidneys of the UUO mice. α-SMA, collagen IV, and fibronectin staining were also more extensive in the kidneys of the UUO mice compared to those of the control mice. The orally administered ω3-PUFAs significantly reduced these immunohistochemically stained areas (Figure 4A,B). The Western blot demonstrated a significant increase in the renal expression of α-SMA, collagen IV, and fibronectin in the kidneys of the UUO mice compared to those of the sham mice. The expression of these proteins was significantly reduced following the treatment with oral ω3-PUFAs (Figure 4C).

### 3.5. Orally Administered ω3-PUFAs Induced Autophagy Flux in the Kidneys of UUO Mice

**Figure 5:**
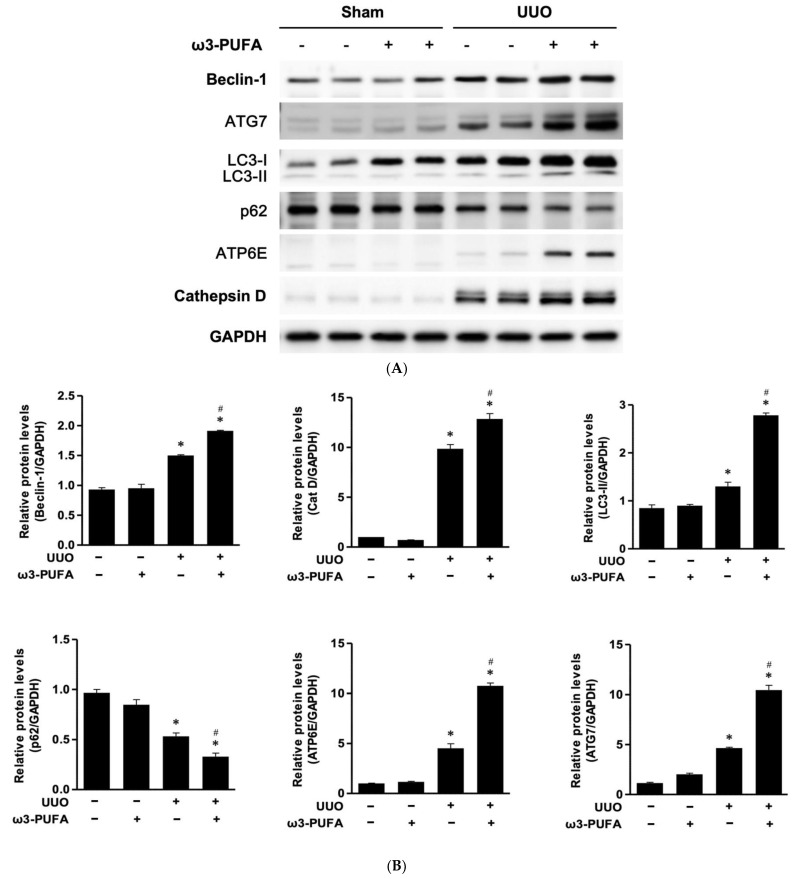
Autophagy activation in the kidneys of mice subjected to UUO and fed ω3-PUFAs. (**A**) Representative Western blot images: the levels of Beclin-1, ATG7, LC3-1, LC3-II, p62, ATP6E, and Cathepsin D were assayed. (**B**) Semiquantitative analysis: expression ratios of Beclin-1, Cathepsin D, LC3-II, p62, ATP6E, and ATG7 to GAPDH, as determined by densitometry. All experiments were performed in triplicate. * *p* < 0.05 vs. sham, # *p* < 0.05 vs. UUO-untreated. PUFAs: polyunsaturated fatty acids; UUO: unilateral ureteral obstruction; GAPDH: glyceraldehyde-3-phosphate dehydrogenase; ATG7: autophagy-related 7; LC3: Microtubule-associated protein 1 light chain 3; ATP6E: V-type proton ATPase subunit E; p62: ubiquitin-binding protein p62.

**Figure 6:**
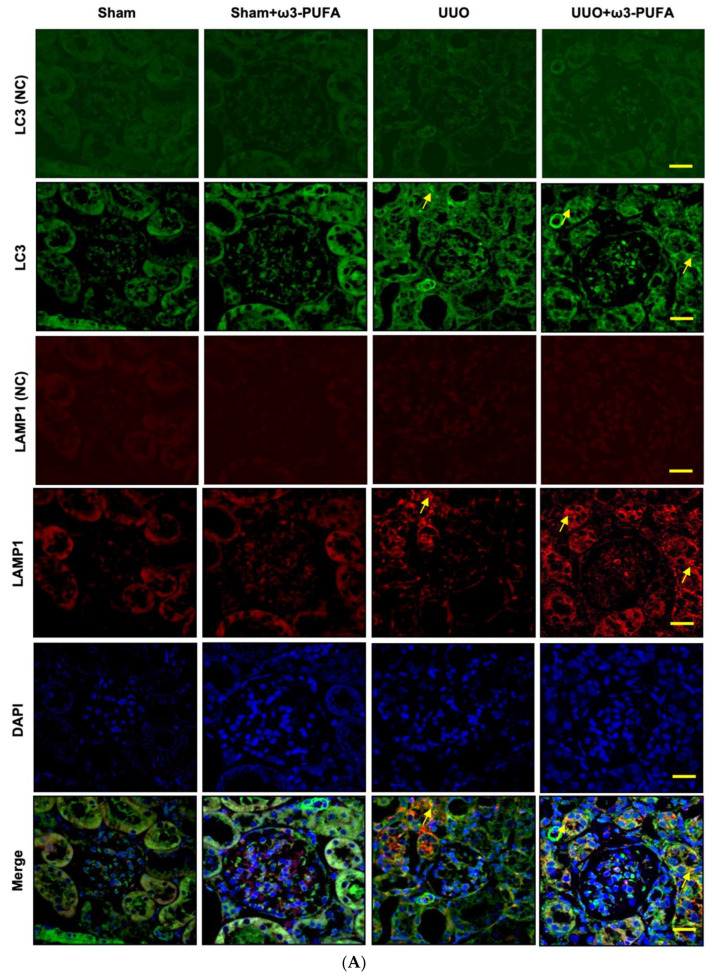

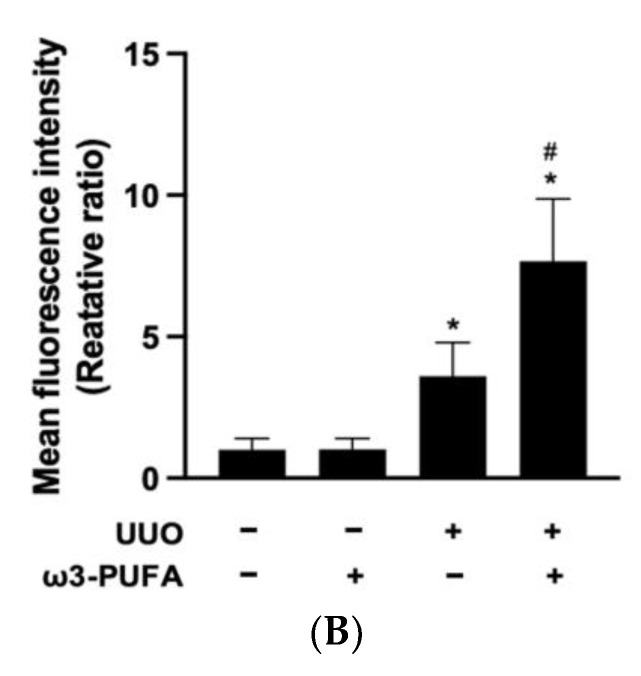
The effects of ω3-PUFAs on autophagy flux in the kidneys of UUO mice, revealed by immunofluorescence staining of LC3 and LAMP1 in proximal tubules. Comparison of four groups: sham mice, ω3-PUFAs-treated sham mice, UUO mice, and ω3-PUFAs-treated UUO mice. (**A**) Representative images for LC3 negative control (NC), LC3, LAMP1 (NC), LAMP1, DAPI, and merged images. LC3 staining (fluorescent green cytoplasmic signal corresponding to proximal tubule cells) in proximal tubule cells was increased in UUO mice, compared to sham mice. In UUO mice treated with ω3-PUFAs, LC3 staining of proximal tubule cells was further increased, compared to that in untreated UUO mice. LAMP1 staining (fluorescent red cytoplasmic signal in proximal tubule cells) of proximal tubule cells was increased in the kidneys of UUO mice compared to those of sham mice. LAMP1 staining in proximal tubule cells was decreased in UUO mice treated with ω3-PUFAs compared to untreated UUO mice. The colocalization of LC3 and LAMP, revealed by the orange fluorescence, indicated appropriate autophagy flux (yellow arrows). (**B**) Quantification of mean fluorescence intensity by ImageJ software bundle with java 8 for Mac OS. The colocalization of LC3 and LAMP1 (orange fluorescence) in the cytoplasm of proximal tubule cells was increased in UUO mice treated with ω3-PUFAs compared to UUO mice. * *p* < 0.05 vs. sham, # *p* < 0.05 vs. UUO-untreated. Original magnification, 400×. Scale bar = 20 μm. PUFAs: polyunsaturated fatty acids; UUO: unilateral ureteral obstruction; LC3: Microtubule-associated protein 1 light chain 3; LAMP: lysosomal associated membrane protein.

The protein expression of Beclin1, ATG7, LC3, ATP6E, Cathepsin D, and p62 was determined to evaluate the autophagy flux in the renal tissue. The protein expression of Beclin1, ATG7, LC3-II, ATP6E, and Cathepsin D was significantly increased in the kidneys of the UUO mice compared to those of the sham mice. However, the orally administered ω3-PUFAs significantly further increased the expression of these proteins in the kidneys of the UUO mice. In contrast, the renal expression of p62 was significantly reduced in the UUO mice compared to the control mice, and the orally administered ω3-PUFAs significantly further reduced it (Figure 5). The co-localization of LC3-II and LAMP-1 was higher in the kidneys of the ω-3 PUFA-treated UUO mice compared to those of the mice subjected to UUO that did not receive ω-3 PUFA (Figure 6).

## 4. Discussion

In this study, we demonstrated that treating UUO mice with ω3-PUFAs enhanced autophagy while attenuating oxidative stress, inflammation, and renal fibrosis. In addition, we showed that two of the main ω3-PUFAs—EPA and DHA—alleviated fibrosis and enhanced autophagy flux in proximal tubular cells.

In the TGF-β1-treated HK2 cells, the DHA/EPA treatment was observed to alleviate the EMT in a dose-dependent manner. In the TGF-β1-treated HK2 cells, the EMT was increased compared to that in the control HK2 cells. At the same time, the levels of LC3-II and p62 were also increased in the TGF-beta-treated HK2 cells compared to the control HK2 cells, indicating that autophagy was initiated, but autophagy flux did not proceed. We found that DHA/EPA alleviated the EMT in a dose-dependent manner. In addition, the DHA/EPA-treated HK2 cells showed a higher expression of LC3-II and a lower expression of p62 in a dose-dependent manner, indicating that autophagy flux proceeded after the treatment with DHA/EPA. Furthermore, DHA/EPA also enhanced AMPK phosphorylation in a dose-dependent manner in the TGF-beta-treated HK2 cells. A previous study conducted by Herzig S. et al. suggested that TGF-beta treatment decreased AMPK phosphorylation and activation, inducing the EMT and myofibroblast activation in proximal tubuloepithelial cells [24]. AMPK is an energy-sensing kinase that plays a crucial role in regulating cell energy homeostasis [25]. The AMPK pathway is activated in unfavorable conditions—such as in the presence of hypoxia, oxidative stress, and nutrient deprivation—and activates catabolic processes, including autophagy [26]. AMPK is considered the main molecular autophagy inducer that counteracts the activity of mTORC1 [27,28,29]. Our past AKI study reported that ω3-PUFAs increased AMPK phosphorylation, enhancing autophagy flux [20]. Similarly, in this study, ω3-PUFAs increased AMPK phosphorylation and promoted autophagy flux in dose-dependent manner. Collectively, EPA and DHA enhanced autophagy by increasing AMPK phosphorylation and boosting autophagy flux, alleviating the EMT.

On the basis of the results of the in vitro experiments reported here, we examined the renoprotective effects of orally administered ω3-PUFAs in UUO mice. A reduction in DNA oxidant damage (as indicated by 8-OHdG staining) and in the number of F4/80-positive cells, also known as macrophage cells, was observed in the UUO mice treated with ω3-PUFAs. The ω3-PUFAs-fed UUO mice also exhibited a reduced expression of osteopontin mRNA and MCP-1 mRNA compared to the control UUO mice (sham mice). The expression of osteopontin and MCP-1 mRNA is associated with macrophage recruitment, and these proteins are considered AKI biomarkers, as reported in other studies [30,31]. Thus, ω3-PUFAs reduced renal oxidative stress and inflammation in the UUO mice. The key feature of tubulointerstitial fibrosis is an excessive deposition of extracellular matrix [2]. The process of EMT transforms the tubular epithelial cells into matrix-forming cells known as myofibroblasts [32]. The renal interstitial fibroblasts and the myofibroblasts produce excessive extracellular matrix (ECM), which subsequently leads to fibrosis in the kidneys [5,32]. Renal fibrosis is a key pathologic mechanism that contributes to the progression of CKD to end-stage renal disease [5]. Masson’s trichrome staining highlights collagen fibers. Fibronectin and collagen IV are ECM proteins, while α-SMA is a marker of mature fibroblasts or myofibroblasts [32]. We performed Masson’s trichrome staining and the immunohistochemical staining of fibronectin and collagen IV and found that ω3-PUFAs inhibited renal fibrosis and EMT, thereby protecting the kidneys and preventing CKD progression.

We examined the expression of beclin-1 and ATG7, which initiate autophagy, as well as that of cathepsin D and ATP6E, which are involved in autophagolysosome maturation, through Western blot analysis. The expression of LC3-II and p62 was also examined for evidence of autophagy flux. Immunofluorescence staining of LC3 and LAMP1 was conducted to examine the effect of ω3-PUFAs on autophagy flux. A previously conducted study demonstrated that autophagy is enhanced in the obstructed proximal tubule following UUO [8,10]. Consistent with this previous study, more extensive autophagy was observed in the kidneys after UUO compared to the sham kidneys. However, the UUO mice fed ω3-PUFAs displayed more extensive autophagic flux in the kidneys compared to the control UUO mice, with lesser oxidative stress, inflammation, and fibrosis.

To the best of our knowledge, this is the only study to show a renoprotective effect of ω3-PUFAs in a UUO model caused by autophagic flux enhancement. Sharma et al. reported a renoprotective effect of ω3-PUFAs in a UUO model as a result of a reduction in EMT regulatory gene expression [33]. Eraky et al. reported that, in rats, ω3-PUFAs protected against acetaminophen-induced hepatic and renal toxicity through the HO-1-Nrf2-BACH1 pathway, mitigating oxidative stress [34]. Yamamoto et al. reported that EPA reduced lipotoxicity in mice administered a high-fat diet and improved autophagy flux [35]. Gwon and Hwang et al. demonstrated the renoprotective effect exerted by omega-3 PUFAs in an I/R (ischemia/reperfusion) renal injury model using fat-1 mice [20].

Other studies have reported the beneficial effects exerted by ω3-PUFAs through autophagy in other organs. Bak et al. demonstrated that ω3-PUFAs prevented streptozotocin-induced Purkinje cell degeneration in fat-1 transgenic mice by enhancing autophagy [36]. A report by Chen et al. indicated that ω3-PUFAs supplementation attenuated traumatic brain injury by inducing autophagy [37]. Another study conducted by Chen et al. reported that ω3-PUFAs protected hepatocytes in the presence of nonalcoholic fatty liver disease through the induction of autophagy [38]. We demonstrated that ω3-PUFAs reduced oxidative injury, inflammation, and fibrosis in UUO mice via autophagic flux enhancement.

Autophagy is a highly conserved degradation system that plays an important role in removing damaged organelles, misfolded protein aggregates, and other macromolecules present in the cytoplasm [39]. In addition, autophagy protects against genome instability and prevents necrosis [40]. Autophagic flux refers to the whole process of autophagy, including autophagosome formation, maturation, fusion with lysosomes, the subsequent breakdown, and the release of macromolecules back into the cytosol [41]. Autophagy flux in damaged kidneys is a protective mechanism with repairing and regeneration effects [42]. In the presence of extensive damage, autophagic repair is not effective, which leads to CKD progression [42]. In addition, autophagy is dysregulated in many kidney diseases, and this contributes to the development of pathophysiological conditions [10,43]. In this scenario, our results are valuable, as we demonstrated that in mice subjected to UUO, ω3-PUFAs enhanced autophagy flux, exerting a renoprotective effect. 

## 5. Conclusions

Our results clearly demonstrated that ω3-PUFAs reduced oxidative stress, inflammation, and renal fibrosis by enhancing autophagy flux in a UUO model of CKD. In vitro, ω3-PUFAs enhanced autophagy flux by increasing AMPK phosphorylation and inhibiting the EMT in HK2 cells. Our study suggests that ω3-PUFAs may provide relief from tubulointerstitial fibrosis in patients with chronic renal disease. It provides insights into the regulation of autophagy, suggesting a new therapeutic option for CKD patients.

## Data Availability

The data presented in this study are available on request from the corresponding author.
